# Efficacy and Safety of TACE Combined With Sorafenib Plus Immune Checkpoint Inhibitors for the Treatment of Intermediate and Advanced TACE-Refractory Hepatocellular Carcinoma: A Retrospective Study

**DOI:** 10.3389/fmolb.2020.609322

**Published:** 2021-01-15

**Authors:** Liyun Zheng, Shiji Fang, Fazong Wu, Weiqian Chen, Minjiang Chen, Qiaoyou Weng, Xulu Wu, Jingjing Song, Zhongwei Zhao, Jiansong Ji

**Affiliations:** Zhejiang Provincial Key Laboratory of Imaging Diagnosis and Minimally Invasive Intervention Research, Lishui Hospital of Zhejiang University, Lishui, China

**Keywords:** TACE-refractory, immune checkpoint inhibitors, sorafenib, transarterial chemoembolization, progression-free survival, overall survival, adverse events, hepatocellular carcinoma

## Abstract

**Purpose:** The study aims to retrospectively investigate the efficacy and safety of sorafenib combined with transarterial chemoembolization (TACE) (TACE+Sor) vs. TACE combined with sorafenib plus immune checkpoint inhibitors (TACE+Sor+ICIs) in treating intermediate and advanced TACE-refractory hepatocellular carcinoma (HCC).

**Materials and Methods:** This study was approved by the ethics committee of Lisui Hospital, Zhejiang University, China. From January 2016 to June 2020, 51 eligible patients with intermediate or advanced TACE-refractory HCC received TACE+Sor (*n* = 29) or TACE+Sor+ICIs (*n* = 22). The differences in tumor response, adverse events (AEs), progression-free survival (PFS), and overall survival (OS) were compared between the two groups. Factors affecting PFS and OS were determined by Cox regression.

**Results:** The disease control rate was higher in the TACE+Sor+ICIs group than in the TACE+Sor group (81.82 vs. 55.17%, *P* = 0.046). Compared with the TACE+Sor group, PFS and OS were prolonged in the TACE+Sor+ICIs group (median PFS: 16.26 vs. 7.30 months, *P* < 0.001; median OS: 23.3 vs. 13.8 months, *P* = 0.012). Multivariate analysis showed that BCLC stage, alpha-fetoprotein and treatment were independent factors of PFS; BCLC, Child-Pugh class, ablation after disease progression and treatment were independent predictive factors of OS. Four patients in the TACE+Sor+ICIs group and three patients in the TACE+Sor group suffered from dose reduction or interruption (18.18 vs. 10.34%, *P* = 0.421). The incidence of ICI-related AEs in the TACE+Sor+ICIs group was well-controlled.

**Conclusion:** The therapeutic schedule of TACE+Sor+ICIs demonstrated efficacy and safety in intermediate and advanced TACE-refractory HCC.

## Introduction

Primary liver cancer (PLC) is a common malignant tumor. Its incidence ranks fifth, with 854,000 new cases per year, making PLC the third leading cause of cancer-related death (Bray et al., 2018; Singal et al., 2020). China is the worst-hit region with a heavy burden of liver cancer. Approximately 364,000 new cases were diagnosed, accounting for half of the new cases of PLC worldwide (Zheng et al., 2018). Hepatocellular carcinoma (HCC) is the most common histological type, accounting for ~75–85% of PLCs. However, ~70% of new patients are diagnosed with intermediate or advanced HCC, missing the opportunity for curative resection (Morise et al., 2014). Moreover, even when patients undergo curative resection, 70% of patients still suffer from recurrence 5 years later (Lacaze and Scotte, 2015; Xiao et al., 2018).

Transarterial chemoembolization (TACE) is currently recommended for intermediate stage of liver cancer and improves the clinical efficacy both before and after curative resection (Sieghart et al., 2015; Wang et al., 2018). According to the Barcelona Clinic Liver Cancer (BCLC) clinical staging system, HCCs in BCLC stage B are recommended for TACE (Han and Kim, 2015; Raoul et al., 2019). In particular, the application of TACE was expanded from BCLC stage A to stage C with Child-Pugh class A or B liver function according to the Chinese guidelines for the diagnosis and treatment of primary liver cancer (2017 edition) (Zhou et al., 2018). Overall, TACE is recommended as the basic therapy for unresectable HCC. However, the efficacy of TACE declines significantly with the number of TACE procedures, with progressive disease (PD) rates of 18, 21, 25, and 27% for the first, second, third, and fourth TACE procedures, respectively (Peck-Radosavljevic et al., 2018). This phenomenon is defined as “TACE failure or refractory,” as proposed by the Japan Society of Hepatology (JSH) and the Liver Cancer Study Group of Japan (LCSGJ) (Kudo et al., 2011, 2014).

The management of advanced TACE-refractory HCC has attracted increasing attention since the concept was proposed by the JSH and LCSGJ in 2014. Efforts have been made to improve the efficacy of TACE by combining with other therapies, including ablation (such as radiofrequency or microwave ablation), radiotherapy (such as stereotactic radiotherapy or radioactive particle seeding), multi-kinase inhibitors (such as sorafenib or apatinib), and immunotherapy (Galle et al., 2017; Guo et al., 2018; Palmer et al., 2020). Sorafenib inhibits tumor angiogenesis by targeting the RAF-MEK-ERK signaling pathway or blocking the expression of vascular endothelial growth factor receptor (VEGFR) and serves as the first line of systemic therapy for HCC (Keating, 2017). Sorafenib can also work as an adjuvant drug for patients diagnosed with advanced HCC (Keating, 2017; Pinyol et al., 2019). Evidence has shown that TACE combined with sorafenib significantly prolongs the recurrence of intermediate or advanced HCC (Kudo et al., 2020). However, data from two phase II/phase III randomized controlled trials (RCTs), the SPACE trial (Lencioni et al., 2016), and the TACE 2 trial (Meyer et al., 2017), failed to demonstrate any clinical benefit from TACE combined with sorafenib. Thus, alternative systemic therapies are urgently needed to improve the patient outcomes of TACE.

Recently, the application of immune checkpoint inhibitors (ICIs) targeting programmed death-1 (PD-1), programmed death-ligand 1 (PD-L1), or cytotoxic T lymphocyte antigen-4 (CTLA-4) has led to a clinical breakthrough for solid tumors (Zhou et al., 2017; Liu and Qin, 2019). Based on a phase I/II clinical trial, CheckMate 040 (El-Khoueiry et al., 2017), the United States Food and Drug Administration (FDA) approved nivolumab for HCC treatment, bringing great promise in restricting tumor recurrence. Due to the need for combinatorial protocols with other antitumor approaches to stimulate the immune system or kill tumor cells directly (Zhou et al., 2017), synergistic combinations with conventional therapies such as radiation, chemotherapy, and targeted therapy have been proposed. Phase II phase III clinical trials of therapies combining ICIs with sorafenib as well as other angiogenesis inhibitors are currently in progress (El Dika et al., 2019).

The hypoxic response induced by TACE boosts the release of proangiogenic cytokines as well as immunogenic cell death (Lencioni, 2012; Huang et al., 2016). These factors further promote tumor angiogenesis and regulate immune function in the tumor microenvironment (Huang et al., 2016; El Dika et al., 2019). Thus, therapy combining TACE with sorafenib plus ICIs may have promising outcomes in intermediate and advanced TACE-refractory HCC. Thus, the aim of this study was to retrospectively compare the efficacy and safety of TACE+sorafenib+ICI treatment with TACE+sorafenib treatment alone in patients with TACE-refractory intermediate and advanced HCC.

## Materials and Methods

### Study Design and Patient Population

**Figure 1:**
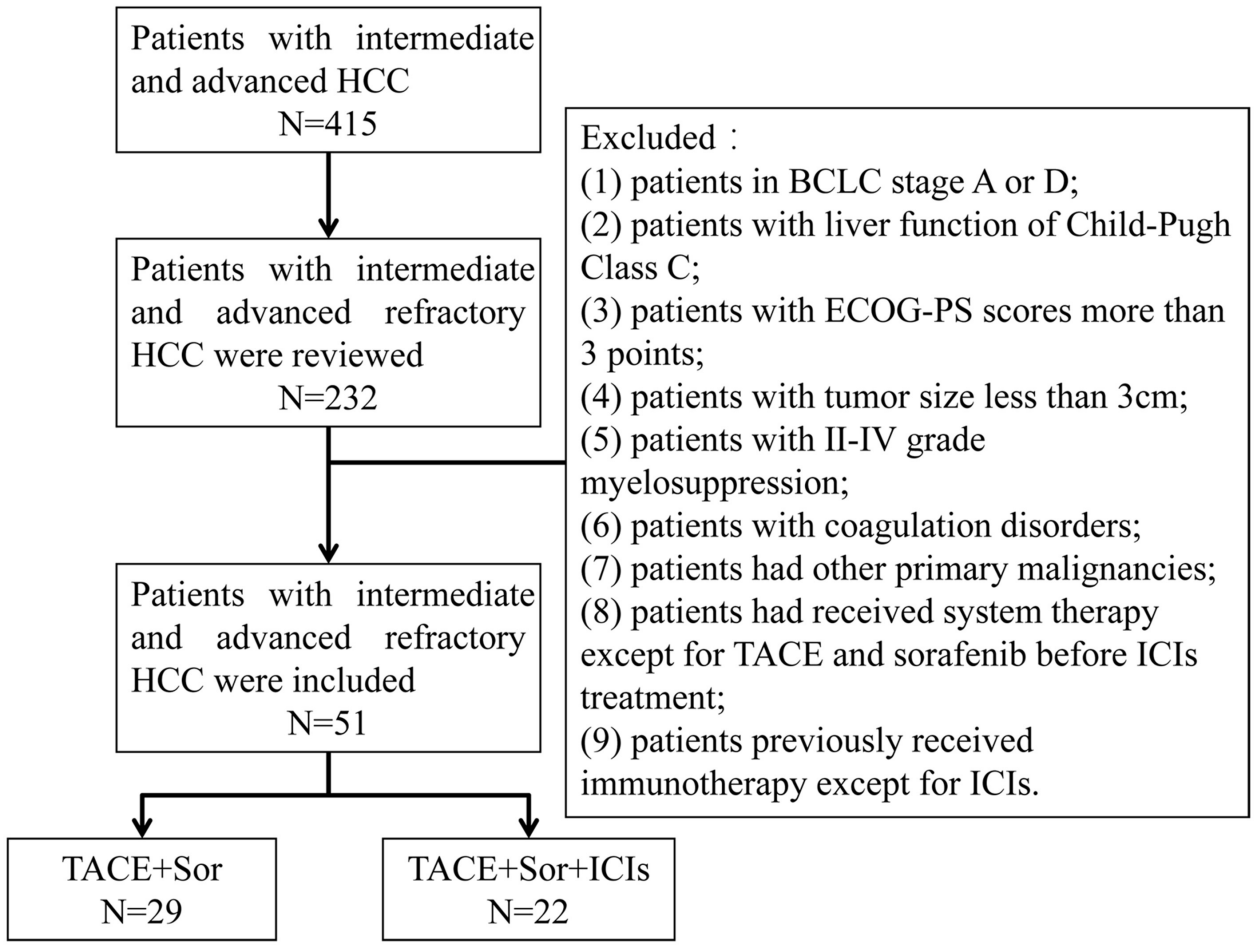
Flowchart shows patient selection. HCC, hepatocellular; BCLC, Barcelona Clinic Liver Cancer; ECOG-PS, Eastern Cooperative Oncology Group performance status; TACE, transarterial chemoembolization; Sor, sorafenib; ICIs, immune checkpoint inhibitors.

Patients diagnosed with intermediate and advanced HCC according to the National Comprehensive Cancer Network (NCCN) Guidelines for Hepatobiliary Cancers were eligible for enrollment (Benson et al., 2019). Candidates from Lishui Hospital of Zhejiang University were enrolled in this retrospective analysis from January 2016 to June 2020. TACE-refractory advanced HCC was determined by the previously published JSH criteria (Kudo et al., 2014). The exclusion criteria were as follows: (1) patients with BCLC stage A or D; (2) patients with Child-Pugh class C liver function; (3) patients with Eastern Cooperative Oncology Group performance status (ECOG-PS) scores was equal to or greater than 3 points; (4) patients with a tumor size <3 cm; (5) patients with grade II-IV myelosuppression; (6) patients with coagulation disorders; (7) patients with other primary malignancies; (8) patients who had received systemic therapy except for TACE and sorafenib before ICI treatment; and (9) patients who previously received immunotherapy except for ICIs (Figure 1).

Based on the above exclusion criteria, 51 eligible patients with TACE-refractory intermediate and advanced HCC with an average age of 56 ± 12.0 years were included in this study, including 46 (90.19%) males and 5 (9.81%) females. The average tumor size was 6.1 ± 2.5 cm, ranging from 3.0 to 12.8 cm. Twenty-three (45.09%) patients with BCLC stage B and 28 (54.91%) patients with BCLC stage B were included. All patients received TACE and sorafenib treatment. Repeated TACE procedures were performed if required after identifying viable lesions or intrahepatic recurrence by MRI imaging. Twenty-nine patients who received TACE plus sorafenib treatment were belong to TACE+Sor group and 22 patients who received TACE plus sorafenib combined with ICIs treatment were served as TACE+Sor+ICIs group. Twelve patients received nivolumab treatment and the other 10 patients selected pembrolizumab treatment in TACE+Sor+ICIs group. This study was approved by the ethics committee of Lishui Hospital, Zhejiang University, China.

### TACE Procedure

TACE was conducted by specialists with more than 10 years of experience in the procedure. In brief, after local anesthesia using 1% lidocaine, the patient was punctured, and an arterial sheath was intubated at the root of the femoral artery by the Seldinger method. Under the guidance of digital subtraction angiography (DSA), a catheter was inserted into the hepatic artery, and a superselective microcatheter was inserted into the feeding artery of the tumors. Oxaliplatin (100–150 mg) and 5-fluorouracil (500–750 mg) were infused though the microcatheter; therefore, the mixed emulsion included 10–30 ml of hyper-liquefying iodide oil, and epirubicin (10–20 mg) was injected into the tumor after hepatic angiography. The exact dose administered to each patient was based on their embolization condition. Absorbable gelatin sponge particles were used to completely embolize the feeding arteries. Finally, iodine tablets were obtained to confirm the complete embolism of the feeding arteries. Repeated TACE would be recommended once the lipiodol deposition shrank and residual lesions occurred, indicating viable lesions or intrahepatic recurrence by contrast-enhanced MRI within 6 weeks after TACE therapy.

### Sorafenib and ICI Administration

The administration of sorafenib and ICIs was initiated within 2 weeks post-TACE therapy based on the proper condition of liver function (aspartate aminotransferase (AST) <40 U/L. Sorafenib at a dose of 400 mg was orally administered twice a day, and intravenous administration of nivolumab or pembrolizumab at a dose of 3 mg/kg was injected every 3 weeks. If patients could not tolerate side effects, dose reduction was determined and recommended by oncologists with more than 10 years of experience. Once serious adverse events (AEs) occur, drug administration cannot be continued.

### Follow-Up and Therapeutic Effect Evaluation

All patients were regularly followed-up and reexamined. The first reexamination of abdominal MRI as well as hematology was conducted within 6 weeks after TACE therapy, and the following reexaminations were recommended every 1–3 months during the treatment. For stable lesions, the time of reexamination was prolonged for 3–6 months. Progression-free survival (PFS) was set as the primary endpoint of this study and was defined as the time interval from TACE refractoriness to the time of disease progression from any cause. The secondary endpoint was overall survival (OS), defined as the period from the time of TACE refractoriness to the time of death. The results of each patient's imaging examinations were evaluated by two diagnostic radiologists with more than 10 years of experience. The efficacy of each therapy was analyzed according to the modified Response Evaluation Criteria in Solid Tumors (mRECIST) as follows. Complete response (CR) was defined as the disappearance of the enhanced tumor area during the arterial phase, meaning complete tissue necrosis. Partial response (PR) was defined as a decrease in the tumor area by at least 30% over a month. PD was defined as an increase of at least 20% in the enhanced tumor area. Stable disease (SD) was defined as neither a sufficient decrease (<30% of the tumor area) nor a sufficient increase in the tumor area (no more than 20% of the tumor area). The overall response rate (ORR) was calculated as (CR+PR)/total number of cases ^*^100%. The formula for calculating the disease control rate (DCR) was (CR+PR+SD)/total number of cases ^*^100%.

### Safety Assessment

AEs were recorded and assessed on the basis of the Common Terminology Criteria for Adverse Events (CTCAE) Version 5.0. According to these criteria, positive AEs were defined as cases with AEs ranked as more than grade 2.

### Statistical Analysis

The analysis of this study was conducted with the statistical software SPSS 24.0(NY Armonk, New York, USA). Continuous variables are expressed as medians and ranges, and categorical variables are expressed as numbers or frequencies. Categorical variables were analyzed using the chi-square test. Continuous variables were analyzed using Student's *t*-test. The survival curves of PFS and OS were analyzed based on the Kaplan–Meier method using the log rank test. Factors with *P* < 0.10 in univariate analysis were further combined into a Cox proportional hazards regression model to identify factors independently associated with PFS and OS. *P* < 0.05 was considered statistically significant.

## Results

### Patient Demographics and Clinical Characteristics

The baseline characteristics, including sex, age, ECOG-PS, HBV infection, cirrhosis, Child-Pugh class, BCLC stage, alpha-fetoprotein (AFP) level, AST level, tumor size, number of tumor nodes, extrahepatic metastasis, portal vein tumor thrombus (PVTT), history of previous surgery, and procedures of TACE, were not significantly different between the two groups (Supplementary Table 1). Herein, the last TACE before refactory is considered to be therapeutic schedule of TACE+systemic therapy (sorafenib with or without presence of ICIs). So the last TACE did not calculated into the previous TACE procedures. A total of 115 previous TACE procedures were performed before TACE refractoriness, with a mean of 2.25 times per patient, including 48 TACE procedures in the TACE+Sor+ICIs group (mean of 2.23 procedures per patient; range 1–4) and 67 TACE procedures in the TACE+Sor group (mean of 1.87 procedures per patient; range 1–4). There was no significant difference between the TACE+Sor+ICIs group and the TACE+Sor group in the number of previous TACE procedures (*F* = 0.049, *P* = 0.82).

### Tumor Response Evaluation

Two patients (9.1%) showed CR, 10 patients (45.5%) showed PR, six patients (27.3%) showed SD, and four patients (18.2%) showed PD in the TACE+Sor+ICIs group. Ten patients (34.5%) showed PR, six patients (20.7%) showed SD, and 13 patients (44.8%) showed PD in the TACE+Sor group. There was a significant difference between the two treatment groups according to mRECIST (*Z* = −2.04, *P* = 0.042) by Mann-Whitney U test. The ORR in the TACE+Sor+ICIs group was significantly higher than that in the TACE+Sor group (54.6 vs. 34.5%, χ^2^ = 2.05, *P* = 0.152). The DCR was significantly different between the two treatment groups (81.8 vs. 55.2%, χ^2^ = 3.99, *P* = 0.046).

### Safety Assessment

Severe AEs (more than grade 4) did not occur among all patients. Common AEs, including decreased appetite, fatigue, and postembolization syndrome (such as nausea or vomiting, fever, and abdominal pain), were found in the early stage of therapy. However, there was no difference between the TACE+Sor+ICIs group and the TACE+Sor group in embolization-related syndrome. AEs related to sorafenib administration, such as hand-foot syndrome, hypertension, alopecia, and diarrhea, occurred, but there was no significant difference between the two groups. The incidences of pruritus and myalgia were higher in the TACE+Sor+ICIs group than in the TACE+Sor group, but there was no significant difference between the two groups. Grade 3–4 rashes occurred in patients who received TACE combined with sorafenib plus ICI therapy, and the incidence was higher than that in patients who received TACE combined with sorafenib alone (3 (13.63%) vs. 0 (0.00%), χ^2^ = 4.20, *P* = 0.040). Fortunately, after receiving glucocorticoid and dose interruptions, the patients recovered within 2 weeks. Even though the incidence rate of hypothyroidism in patients who received TACE combined with sorafenib plus ICI therapy was higher than that in patients who received TACE combined with sorafenib alone [2 (9.09%) vs. 0 (0.00%), χ^2^ = 2.74, *P* = 0.098], the difference was not significant. Proteinuria, hypokalemia, increased AST, granulocytopenia, decreased neutrophil count, and hyperbilirubinemia occurred in both groups, but there were no significant differences between the two groups.

### Follow-Up Treatment After Tumor Progression

As shown in Supplementary Table 3, follow-up treatments, including ablation, radiotherapy, and second-line anti-angiogenesis agents, were applied in both groups. No significant difference was found in the follow-up treatment after disease progression between the two groups.

### Comparison of PFS and OS Between the Two Groups

**Figure 2:**
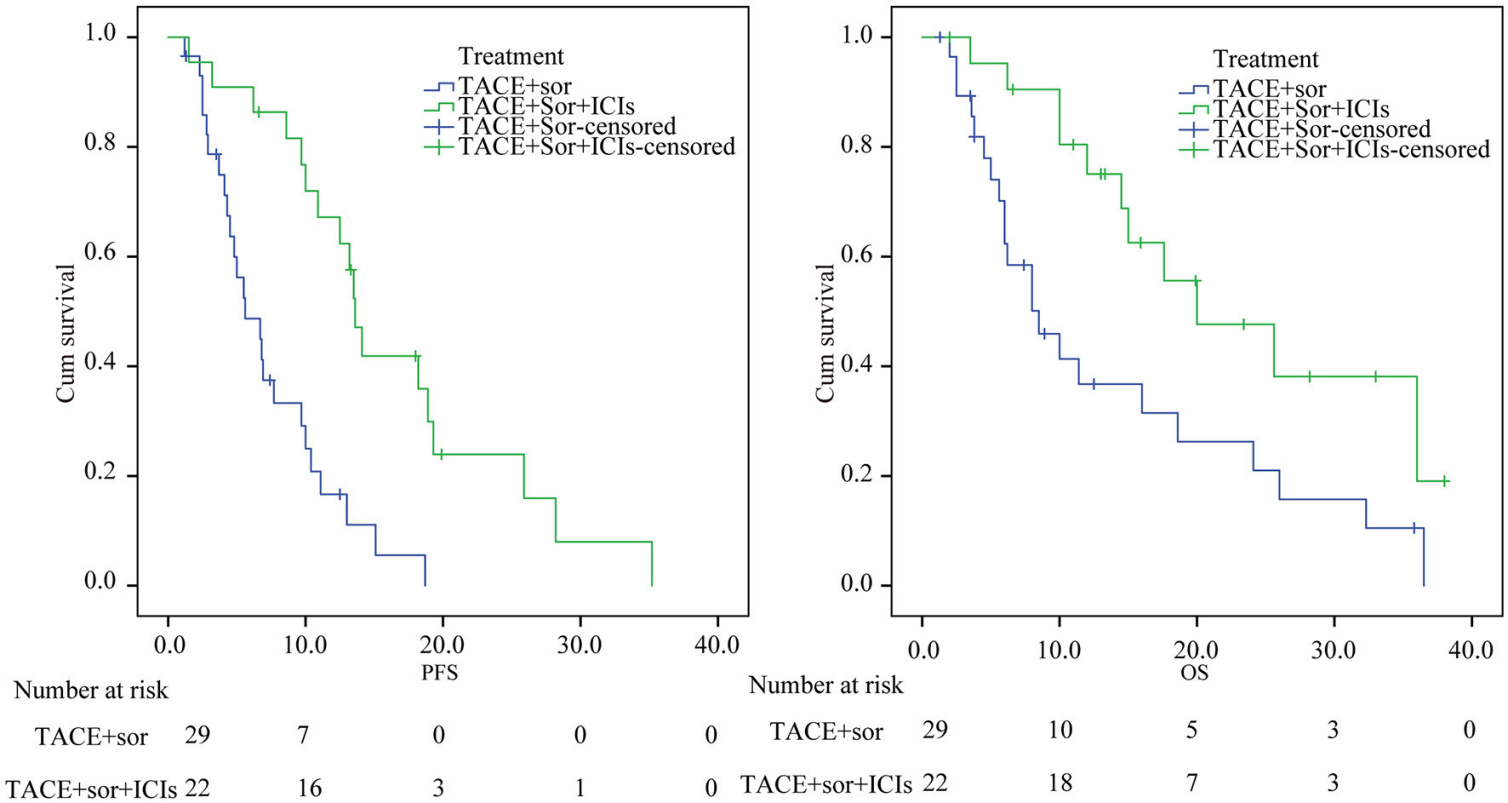
Kaplan–Meier analysis of PFS and OS. TACE, transarterial chemoembolization; Sor, sorafenib; ICIs, immune checkpoint inhibitors; PFS, Progression-free survival; OS, overall survival.

The median PFS was 16.26 (95% confidence interval [CI] 12.1–20.37) months in the TACE+Sor+ICIs group, while the median PFS was 7.30 (95% CI 5.49–9.12) months in the TACE+Sor group. Compared with the TACE+Sor group, the OS of the TACE+Sor+ICIs group was significantly longer (log rank test, *z* = 15.5, *P* < 0.001, Figure 2). The half-year, 1-year, and 2-year PFS rates were 90.09, 67.59, and 42.56% in the TACE+Sor+ICIs group and 50.0, 18.0, and 7.7 in the TACE+Sor group. The median OS was 13.8 (95% CI 9.11–18.50) months in the TACE+Sor group and 23.3 (95% CI 17.56–29.07) months in the TACE+Sor+ICIs group. Compared with the TACE+Sor group, the OS of the TACE+Sor+ICIs group was significantly longer (log rank test, *z* = 6.31, *P* = 0.012, Figure 2). The 1-year, 2-year, and 3-year survival rates were 80, 48.2, and 36.2% in the TACE+Sor+ICIs group and 40.0, 29.1, and 9.7% in the TACE+Sor group. In addition, the median PFS of patients receiving nivolumab was comparable to those receiving pembrolizumab [13.6 months (95% C12.02–15.18) vs. 13.2 months (95% CI 6.85–19.55, *z* = 0.32, *P* = 0.859] in the TACE+Sor+ICIs group. And the median OS of patients selecting nivolumab treatment was similar to those receiving pembrolizumab [20.0 months (95% C12.92–27.08) vs. 25.6 months (95% CI 13.53–37.67, *z* = 0.05, *P* = 0.820] in the TACE+Sor+ICIs group (seen in Supplementary Figure 1).

### Prognostic Factors Affecting OS and PFS

The univariate analysis results of the factors influencing PFS are shown in Supplementary Table 2. The data indicated that sex, age, ECOG-PS, HBV infection, cirrhosis, AST, tumor node, surgery history, and number of previous TACE procedures were not factors associated with PFS (*P* > 0.10). Factors with *P* < 0.10 including Child-Pugh class, BCLC stage, AFP, tumor size, metastasis, PVTT, and treatment were combined into the Cox proportional hazards regression model. Multivariate analysis showed that BCLC stage (C vs. B) (hazard ratio [HR] = 2.14; 95% CI: 1.01–4.51; *P* = 0.047), AFP level (≥400 ng/mL vs. <400 ng/mL) (HR = 2.20; 95% CI: 1.08–4.78; *P* = **0.048**), tumor size (≥5 cm vs. <5 cm) (HR = 3.25; 95% CI: 1.47–7.19; *P* = 0.003) and treatment (TACE+Sor+ICI treatment vs. TACE+Sor treatment) (HR = 0.11; 95% CI: 0.05–0.26; *P* < 0.001) were independent predictive factors of PFS. Moreover, univariate analyses showed that sex, age, ECOG-PS, HBV infection, cirrhosis, AST, tumor node, surgery history, and number of previous TACE procedures were not factors associated with OS. Multivariate analysis indicated that Child-Pugh class (B vs. A) (HR = 2.36; 95% CI, 1.02–5.46; *P* = 0.044), BCLC stage (C vs. B) (HR = 3.88; 95% CI: 1.56–9.60; *P* = 0.003), treatment (TACE+Sor+ICI treatment vs. TACE+Sor treatment) (HR = 0.24; 95% CI: 0.11–0.55; *P* = 0.001), and ablation after disease progression (yes vs. no) (HR = 0.29; 95% CI: 0.12–0.75; *P* = 0.010) were independent predictive factors of OS.

### Subgroup Analysis

**Figure 3:**
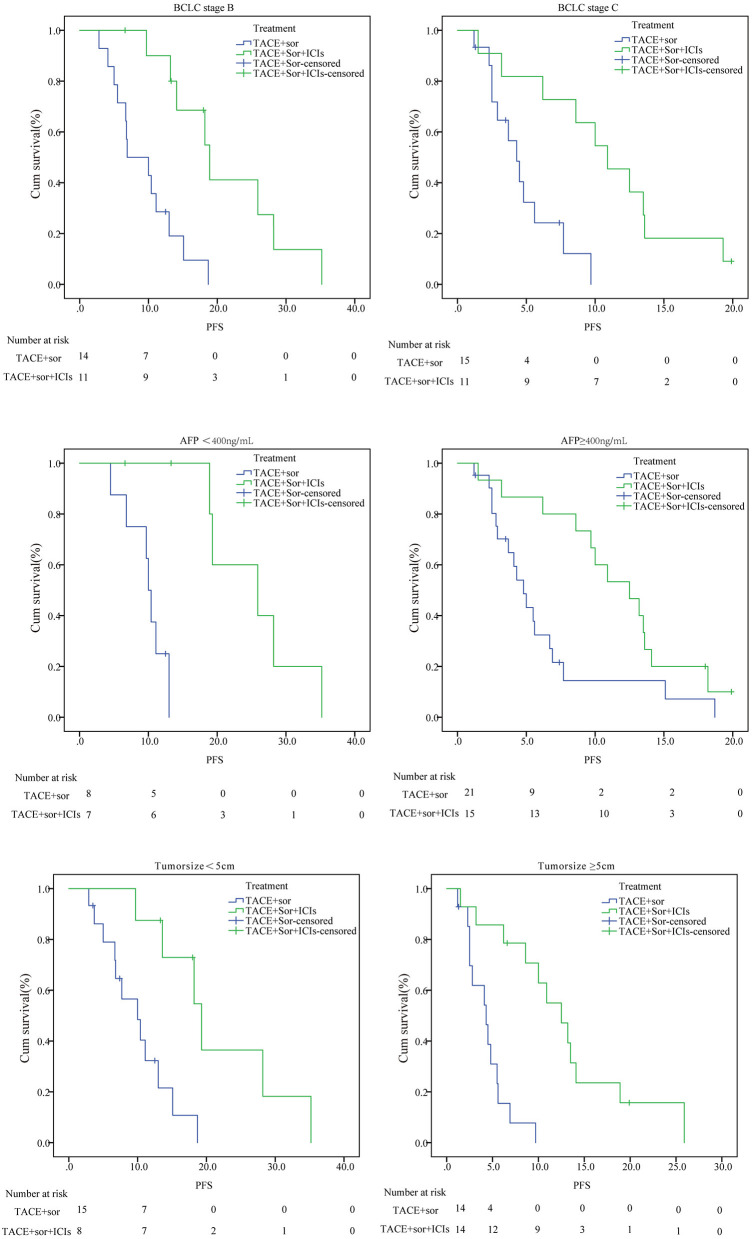
Subgroup analysis of PFS. TACE, transarterial chemoembolization; Sor, sorafenib; ICIs, immune checkpoint inhibitors; PFS, Progression-free survival; OS, overall survival.

**Figure 4:**
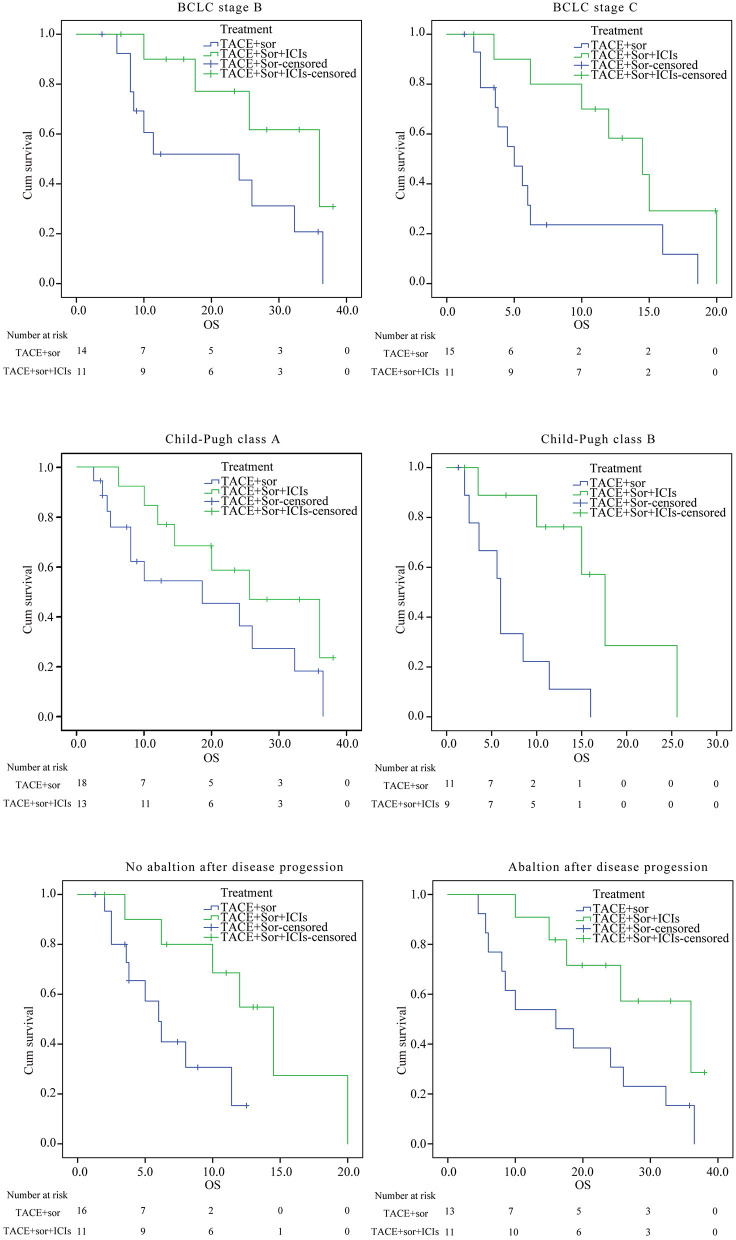
Subgroup analysis of OS. BCLC, Barcelona Clinic Liver Cancer; AFP, alpha-fetoprotein; TACE, transarterial chemoembolization; Sor, sorafenib; ICIs, immune checkpoint inhibitors; PFS, Progression-free survival; OS, overall survival.

As seen in figures 3 and 4, in patients with BCLC stage B, the median PFS was 9.4 months (95% CI: 6.8–11.9) in the TACE+Sor group and 21.2 months (95% CI: 15.5–27.00) in the TACE+Sor +ICIs group (log rank = 11.73, *P* = 0.001). The corresponding OS was 20.3 months (95% CI: 13.3–27.4) in the TACE+Sor group and 30.0 months (95% CI: 23.5–36.6) in the TACE+Sor+ICIs group (log rank = 3.30, *P* = 0.069). In patients with BCLC stage C, the median PFS was 4.8 months (95% CI: 3.3–6.2) in the TACE+Sor group and 10.8 months (95% CI: 7.5–14.1) in the TACE+Sor+ICIs group (log rank = 9.08, *P* = 0.003). The corresponding OS was 7.3 months (95% CI: 4.0–10.5) in the TACE+Sor group and 13.5 months (95% CI: 9.7–17.3) in the TACE+Sor+ICIs group (log rank = 4.85, *P* = 0.028). In patients with AFP levels lower than 400 ng/mL, the median PFS was 9.8 months (95% CI: 7.8–11.8) in the TACE+Sor group and 25.5 months (95% CI: 19.6–31.4) in the TACE+Sor+ICIs group (log rank = 10.63, *P* = 0.001). In patients with AFP levels higher than 400 ng/mL, the median PFS was 6.2 months (95% CI: 3.9–8.5) in the TACE+Sor group and 11.6 months (95% CI: 8.9–14.2) in the TACE+Sor+ICIs group (log rank = 5.92, *P* = 0.015). In patients with tumor diameters <5 cm, the median PFS was 10.0 months (95% CI: 7.3–12.6) in the TACE+Sor group and 21.6 months (95% CI: 14.4–28.8) in the TACE+Sor+ICIs group (log rank = 8.50, *P* = 0.004). In patients with tumor diameters >5 cm, the median PFS was 4.4 months (95% CI: 3.1–5.6) in the TACE+Sor group and 12.8 months (95% CI: 8.8–16.9) in the TACE+Sor+ICIs group (log rank = 16.64, *P* = 0.000). In patients with Child-Pugh class A, the median OS was 18.6 months (95% CI: 11.8–25.4) in the TACE+Sor group and 25.7 months (95% CI: 19.0–32.4) in the TACE+Sor+ICIs group (log rank = 3.37, *P* = 0.066). In patients with Child-Pugh class B, the median OS was 6.8 months (95% CI: 3.9–9.8) in the TACE+Sor group and 14.1 months (95% CI: 10.1–18.0) in the TACE+Sor+ICIs group (log rank = 6.61 *P* = 0.010). Among patients who did not receive ablation after disease progression, the median OS was 6.9 months (95% CI: 4.8–8.9) in the TACE+Sor group and 13.2 months (95% CI: 9.0–17.4) in the TACE+Sor+ICIs group (log rank = 4.81, *P* = 0.028). Among patients who received ablation after disease progression, the median OS was 17.9 months (95% CI: 11.3–24.4) in the TACE+Sor group and 28.9 months (95% CI: 22.6–35.3) in the TACE+Sor+ICIs group (log rank = 4.08, *P* = 0.043).

## Discussion

With a decade of drug development, the therapeutic reversal of immune exhaustion by ICIs has been shown to be effective in advanced HCC. Several published reports have indicated that ICIs elevated the tumor response and prolonged the time to recurrence as well as the OS of advanced HCC. The Check Mate-040 trial showed that patients can clinically benefit from nivolumab at a dose of 3 mg/kg (El-Khoueiry et al., 2017). Like nivolumab, pembrolizumab prolonged the median PFS up to 4.9 months and the median OS up to 12.9 months in advanced HCC patients receiving sorafenib frontline in the KEYNOTE-224 trial (Zhu et al., 2018).

The combination of ICIs and antiangiogenic agents was proposed by investigators due to the additional immunomodulatory effects of antiangiogenic agents (Hilmi et al., 2019). The preliminary results showed that lenvatinib plus pembrolizumab for advanced HCC showed good toxicity tolerance and an objective response rate of 48% (Makker et al., 2017). Other ongoing studies are also focusing on the efficacy and safety of combination therapy, such as nivolumab plus sorafenib or lenvatinib and pembrolizumab plus regorafenib. Thus, the combination of ICIs and sorafenib may be a potential therapy for advanced HCC.

Studies have demonstrated that TACE activates a hypoxic response, promoting the release of vascular endothelial growth factor (VEGF) and other proangiogenic cytokines (Sergio et al., 2008; Viveiros et al., 2019). Additionally, cell necrosis induced by TACE is predicted to be associated with antigen release and the exposure of damage-associated molecular patterns. The application of ICIs can bolster the function of T cells and antigen-presenting cells (APCs) after TACE plus sorafenib therapy. The treatment of TACE combined with sorafenib plus ICIs may obtain a better clinical effect for intermediate and advanced HCC by enabling a robust tumor-specific immune response and inhibiting tumor angiogenesis. In this study, our results revealed that patients who received TACE combined with sorafenib plus ICIs had prolonged OS and PFS compared with those who only received TACE combined with sorafenib alone (median OS: 23.3 vs. 13.8 months, log rank = 6.31, *P* = 0.012; median PFS: 16.26 vs. 7.31 months, log rank = 15.48, *P* = 0.000). As reported by the TACTICS trial, the median PFS of patients who received TACE plus sorafenib was up to 25.2 months, which was much higher than that of patients who received TACE combined with sorafenib plus ICIs or TACE combined with sorafenib alone. The reason is that new intrahepatic lesions were not regarded as PD in the TACTICS trial (Kudo et al., 2020).

Studies have demonstrated that TACE combined with sorafenib or other antineoplastic agents is an independent predictor of prognosis for intermediate and advanced HCC (Wu et al., 2017; Takada et al., 2019; Kudo et al., 2020). In this study, the treatment of TACE combined with sorafenib plus ICIs was a protective factor for PFS and OS in intermediate and advanced refractory HCC patients. The BCLC stage (C vs. B) was an independent predictive factor for PFS and OS. AFP level (≥400 vs. <400 ng/mL) and tumor size (≥5 vs. <5 cm) were risk factors for PFS. Child-Pugh class (B vs. A) was an independent predictive factor for OS. Studies have suggested that other factors, such as AST level, number of nodules, vascular invasion, and metastasis, are also significant predictors of OS or PFS (Peng et al., 2018; Takada et al., 2019). Our data showed that PVTT and metastasis significantly affected OS or PFS in univariate analyses but were adjusted in multivariate analysis. This may be due to the cooperation of PVTT and metastasis in BCLC stage. The small sample size and relatively short follow-up time may also be other reasons. Our data also showed that follow-up treatment was a protective factor for OS after disease progression of intermediate and advanced HCC. The median OS of patients who received ablation was higher than that of patients who did not receive ablation. Among patients who received ablation after disease progression, the median OS of patients treated with TACE combined with sorafenib plus ICIs was higher than that of patients treated with TACE combined with sorafenib alone. These results indicated that ablation after disease progression prolongs the OS of advanced HCC. In addition, our results suggested that patients who received radiotherapy or second-line antiangiogenesis agents had a longer OS. However, the results may also be caused by selection bias, since selected patients who received following treatment tended to have a good performance.

The AEs in this study were mild to moderate and could easily be controlled. The incidences of postembolization syndrome, such as nausea, vomiting, fever, and abdominal pain, and sorafenib-related AEs, such as hand-foot syndrome, hypertension, diarrhea, and alopecia, were similar to those in previous studies of patients treated with TACE combined with sorafenib and TACE or sorafenib alone (Lencioni et al., 2016; Meyer et al., 2017; Ye et al., 2018; Kudo et al., 2020). The incidence of dose reductions or interruptions between the two groups was not significantly different. Three patients who received TACE combined with sorafenib alone suffered from dose reductions or interruptions owing to increased AST levels caused by sorafenib administration. There were four patients who received TACE combined with sorafenib plus ICIs who interrupted sorafenib and ICI administration due to increased aspartate aminotransferase levels (two patients), hypothyroidism (one patient), and rash (one patient). Fortunately, after dose reductions or interruptions and hepatinica, thyroxine or glucocorticoid, all patients returned to normal.

This study indicated that patients who received the combination of TACE with sorafenib plus ICIs had promising outcomes. However, some limitations must be considered. As a retrospective study, it has all of the defects inherent to this type of study design. For example, the background of patients, including financial capability, education, and cognition of liver cancer, have an impact on the choice of therapy by patients and physicians. In addition, the limitation of the sample size of patients and the length of follow-up also had a significant impact on the outcome.

In conclusion, the combination TACE with sorafenib plus ICIs prolongs the PFS and OS of intermediate and advanced refractory HCC patients. TACE with sorafenib plus ICI treatment was a protective predictive factor for PFS, while BCLC stage, AFP level, and tumor size were poor predictive factors for PFS. Child-Pugh class, AFP level, BCLC stage, TACE with sorafenib plus ICI treatment, and follow-up ablation were independent predictive factors for OS. Severe AEs rarely occurred, and we confirmed the clinical safety of using TACE with sorafenib plus ICI treatment. Overall, it is efficient and safe for patients with intermediate and advanced refractory HCC to receive TACE with sorafenib plus ICI therapy.

## Data Availability Statement

The raw data supporting the conclusions of this article will be made available by the authors, without undue reservation.

## Ethics Statement

The studies involving human participants were reviewed and approved by Ethics Committee of Lishui hospital Zhejiang University. The patients/participants provided their written informed consent to participate in this study. Written informed consent was obtained from the individual(s) for the publication of any potentially identifiable images or data included in this article.

## Author Contributions

ZZ and JJ designed the research. LZ and SF carried out the study and wrote the first draft of the manuscript. FW and WC helped in the study. XW helped in analyzing the data. MC and QW helped in manuscript writing, editing, and critical evaluation. JJ supervised the project and read and approved the manuscript. All authors contributed to the article and approved the submitted version.

## Conflict of Interest

The authors declare that the research was conducted in the absence of any commercial or financial relationships that could be construed as a potential conflict of interest.
