# Supplementary figures and images for: Efficacy and Safety of TACE Combined With Sorafenib Plus Immune Checkpoint Inhibitors for the Treatment of Intermediate and Advanced TACE-Refractory Hepatocellular Carcinoma: A Retrospective Study
Source: Front Mol Biosci. 2021 Jan 15;7:609322. doi: 10.3389/fmolb.2020.609322 (PMC7843459; doi:10.3389/fmolb.2020.609322)

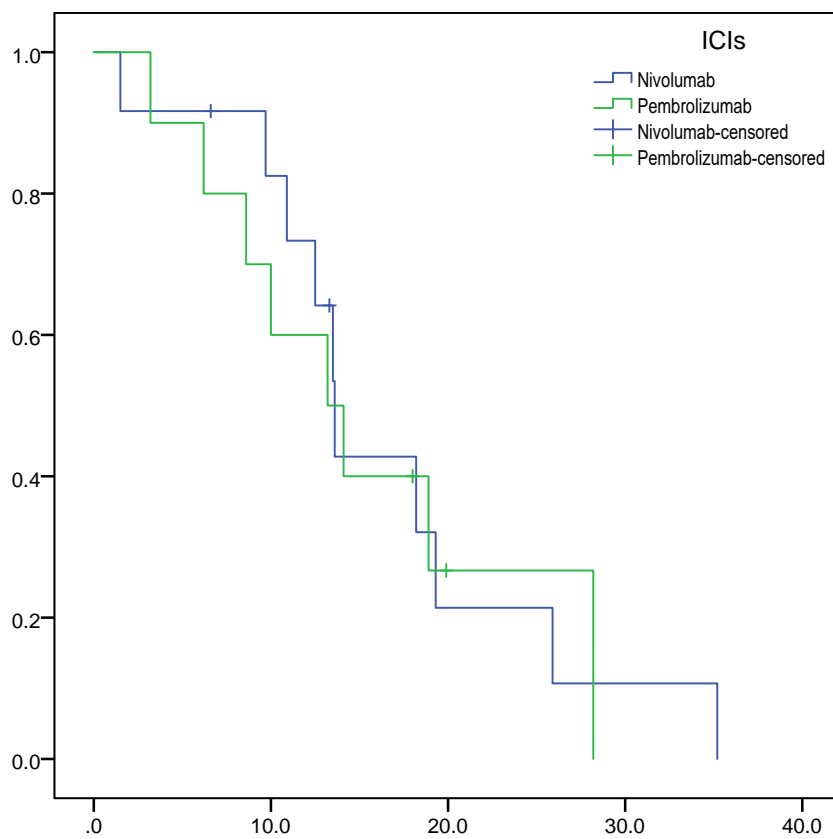

Number at risk

|               |    |   |   |   |   |
|---------------|----|---|---|---|---|
| Nivolumab     | 12 | 9 | 2 | 1 | 0 |
| Pembrolizumab | 10 | 7 | 1 | 0 | 0 |

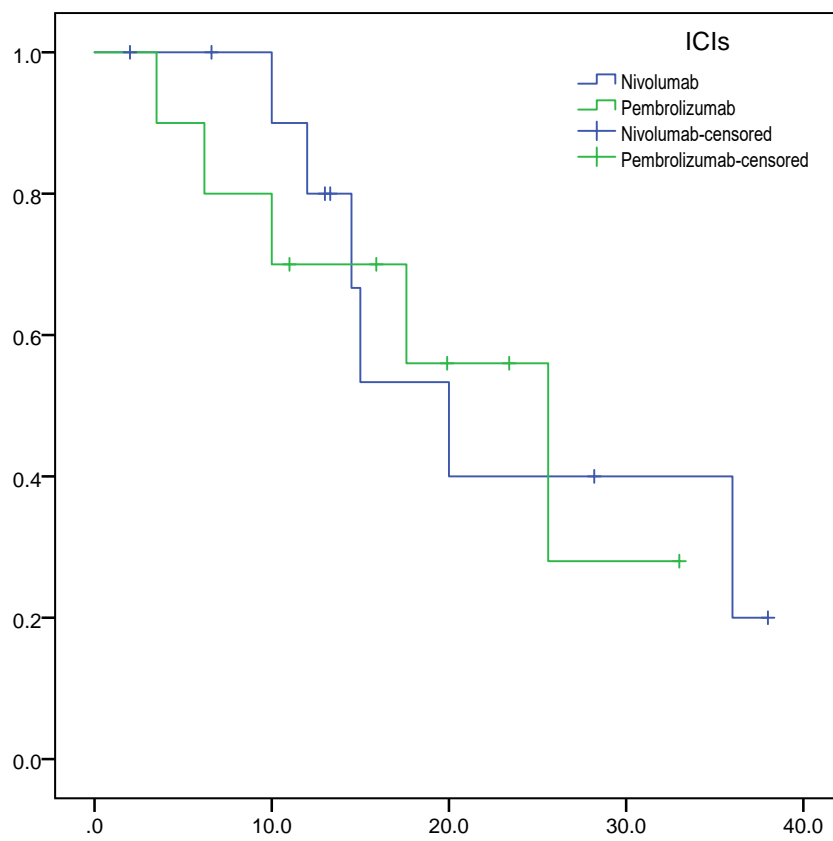

Number at risk

|               |    |    |   |   |   |
|---------------|----|----|---|---|---|
| Nivolumab     | 12 | 10 | 4 | 2 | 0 |
| Pembrolizumab | 10 | 8  | 3 | 1 | 0 |

Supplement: Supplementary file 1 [file Data_Sheet_1.PDF]
