# Supplementary material for: Efficacy and Safety of TACE Combined With Sorafenib Plus Immune Checkpoint Inhibitors for the Treatment of Intermediate and Advanced TACE-Refractory Hepatocellular Carcinoma: A Retrospective Study
Source: Front Mol Biosci. 2021 Jan 15;7:609322. doi: 10.3389/fmolb.2020.609322 (PMC7843459; doi:10.3389/fmolb.2020.609322)
Supplement: Supplementary file 2 [file Table_1.DOCX]

Table 1 Patient demographics and clinical characteristics

| Characteristics | Total(n=51) | TACE+Sor group（n=29） | TACE+Sor+ICIs group（n=22） | Test value | *P value* |
| --- | --- | --- | --- | --- | --- |
| Sex |  |  |  | 0.12 | 0.726 |
| Male | 43(84.31%) | 24(82.75%) | 19(86.36%) |  |  |
| Female | 8(9.81%) | 5(17.25%) | 3(13.64%) |  |  |
| Age（years） |  |  |  | 1.40 | 0.238 |
| ＜55 | 28(54.90%) | 18(62.06%) | 10(45.45%) |  |  |
| ≥55 | 23(45.10%) | 11(37.94%) | 12(54.55%) |  |  |
| ECOG-PS |  |  |  | 1.40 | 0.237 |
| 0-1 | 21(41.18%) | 14(48.27%) | 7(31.82%) |  |  |
| 2 | 30(58.82%) | 15(51.73%) | 15(68.18%) |  |  |
| HBV infection |  |  |  | 2.65 | 0.104 |
| Postive | 44(86.27%) | 27(93.1%) | 17(77.27%) |  |  |
| Negtive | 7(13.73%) | 2(6.9%) | 5(22.73%) |  |  |
| Cirrosis |  |  |  | 0.91 | 0.343 |
| Yes | 36(70.59%) | 22(75.86%) | 14(63.64%) |  |  |
| No | 15(29.41%) | 7(24.14%) | 8(36.36%) |  |  |
| Child-Pugh |  |  |  | 0.05 | 0.829 |
| A | 31(60.78%) | 18(62.07%) | 13(59.03%) |  |  |
| B | 20(39.22%) | 11(37.93%) | 9 (40.97%) |  |  |
| BCLC stage |  |  |  | 0.02 | 0.903 |
| B | 25(52.94%) | 14(49.02%) | 11(50.00%) |  |  |
| C | 26(47.06%) | 15 (50.98%) | 11(50.00%) |  |  |
| AFP（ng/ml） |  |  |  | 0.11 | 0.743 |
| ＜400 | 15(33.33%) | 8(25.59%) | 7(31.82%) |  |  |
| ≥400 | 36(66.67%) | 21(74.41%) | 15(68.18%) |  |  |
| AST（U/L） |  |  |  | 0.37 | 0.543 |
| ≤40 | 30(58.82%) | 16(55.18%) | 14(63.64%) |  |  |
| ＞40 | 21(41.18%) | 13(44.82%) | 8(36.36%) |  |  |
| Tumor size |  |  |  |  |  |
| ＜5cm | 23(45.09%) | 15(51.72%) | 8(36.36%) | 1.19 | 0.275 |
| ≥5cm | 28(54.91%) | 14(48.28%) | 14(63.64%) |  |  |
| Tumor node |  |  |  |  |  |
| Single | 16(31.37%) | 9(31.03%) | 7(31.82%) | 0.00 | 0.952 |
| Multiple | 68.6(68.63%) | 20(68.97%) | 15(68.18%) |  |  |
| PVTT |  |  |  | 0.11 | 0.743 |
| Yes | 15(29.42%) | 8(25.59%) | 7(31.82%) |  |  |
| No | 36(70.58%) | 21(74.41%) | 15(68.18%) |  |  |
| Metastasis |  |  |  | 0.89 | 0.346 |
| Yes | 20(39.22%) | 13(44.83%) | 7(31.82%) |  |  |
| No | 31(60.78%) | 16(55.17%) | 15(68.18%) |  |  |
| Surgery |  |  |  |  |  |
| Yes | 11(21.57%) | 5(17.25%) | 6(27.28%) | 0.11 | 0.74 |
| No | 40(78.43%) | 24(82.75%) | 16(72.72%) |  |  |
| Number of previous TACE |  |  |  |  |  |
| ≤2 | 30(58.82%) | 18(62.07%) | 12(54.54%) | 0.29 | 0.59 |
| ＞2 | 21(41.18%) | 11(37.93%) | 10(45.46%) |  |  |

Abbreviations: TACE, transarterial chemoembolization; Sor, sorafenib; ICIs，immune checkpoint inhibitors; ECOG-PS, Eastern Cooperative Oncology Group performance status; BCLC, Barcelona Clinic Liver Cancer; AFP, alpha-fetoprotein; AST, aspartate aminotransferase e; PVTT, portal vein tumor thrombus. Test value and *P* value are obtained by comparing the difference between the two groups.

Table 2 Tumor Response

|  | CR | PR | SD | PD | ORR | DCR |
| --- | --- | --- | --- | --- | --- | --- |
| TACE+Sor | 0(0.00%) | 10(34.48%) | 6(20.69%) | 13(44.83%) | 10(34.48%) | 16(55.17%) |
| TACE+Sor+ICIs | 2(9.09%) | 10(45.45%) | 6(27.27%) | 4(18.18%) | 12(54.55%) | 18(81.82%) |
| Test value | -2.04 | | | | 2.05 | 3.99 |
| P value | 0.042 | | | | 0.152 | 0.046 |

Abbreviations: CR, complete response; PR, partial response; SD, stable disease; PD, progressive disease; ORR, overall response rate; DCR, disease control rate.

Table 3 Treatment-related Adverse Events

|  | TACE+Sor group（n=29） | TACE+Sor+ICIs group（n=22） | Test value | *P value* |
| --- | --- | --- | --- | --- |
| Dose reduce or interruptions | 3(10.34%) | 4(18.18%) | 0.65 | 0.421 |
| Decreased appetite | 8 (27.58%) | 10(45.45%) | 1.75 | 0.186 |
| Fatigue | 9(31.03%) | 10(45.45%) | 1.11 | 0.291 |
|  |  |  |  |  |
| Fever | 10(34.48%) | 7(31.82%) | 0.04 | 0.842 |
| Nausea or vomiting | 11(37.93%) | 7(31.82%) | 0.20 | 0.651 |
| Abdominal pain | 9(31.03%) | 8(36.36%) | 0.16 | 0.689 |
|  |  |  |  |  |
| Hand and foot syndrome | 8(27.58%) | 8(36.36%) | 0.45 | 0.503 |
| Hypertension | 4(13.79%) | 7(31.82%) | 2.40 | 0.121 |
| Alopecia | 2(6.89%) | 4(18.18%) | 1.53 | 0.215 |
| Diarrhoea | 1(3.44%) | 3(13.63%) | 1.79 | 0.180 |
|  |  |  |  |  |
| Pruritus | 4(13.79%) | 5(22.72%) | 0.68 | 0.407 |
| Myalgia | 1(3.44%) | 4(18.18%) | 3.07 | 0.08 |
| Rash | 0(0.00%) | 3(13.63%) | 4.20 | 0.040 |
| Hypothyroidism | 0(0.00%) | 2(9.09%) | 2.74 | 0.098 |
|  |  |  |  |  |
| Proteinuria | 5(17.24%) | 9 (36.36%) | 2.41 | 0.121 |
| Hypokalemia | 3(10.34%) | 5(22.72%) | 1.45 | 0228 |
| Increased aspartate aminotransferase | 3(10.34%) | 4(18.18%) | 0.65 | 0.421 |
| Decreased neutrophil count | 3(10.34%) | 4(18.18%) | 0.65 | 0.421 |
| Hyperbilirubinemia | 5(17.24%) | 7(31.82%) | 1.47 | 0.224 |

Table 4 follow-up treatments after disease progression

|  | Total(n=51) | TACE+Sor group（n=29） | TACE+Sor+ICBs group（n=22） | Test value | P value |
| --- | --- | --- | --- | --- | --- |
| Ablation |  |  |  | 0.13 | 0.714 |
| Yes | 23(45.10%) | 13(55.17%) | 11(50.00%) |  |  |
| No | 28(54.90%) | 16(45.93%) | 11(50.00%) |  |  |
| Radiotherapy |  |  |  | 1.44 | 0.23 |
| Yes | 11(21.57%) | 8(27.59%) | 3(13.63%) |  |  |
| No | 40(78.43%) | 21(72.41%) | 19(86.37%) |  |  |
| Second-line anti-angiogenesis agents |  |  |  |  |  |
| Yes | 27(52.94%) | 17(58.62%) | 10(45.45%) | 0.87 | 0.351 |
| No | 24(47.06%) | 12(42.38%) | 12(54.55%) |  |  |

Table 5 Univariate and multivariate analyses of prognostic factors affecting PFS in TACE-refractory advanced HCC

| Variables | Univariate Cox analysis | | |  | Multivariate Cox analysis | | |
| --- | --- | --- | --- | --- | --- | --- | --- |
|  | HR | 95%*CI* | *P* value | | HR | 95%*CI* | *P* value |
| Sex(male vs female) | 1.32 | 0.58-3.00 | 0.508 | |  |  |  |
| Age(＜55 vs ≥55) | 1.17 | 0.64-2.16 | 0.612 | |  |  |  |
| ECOG-PS (0-1 vs 2) | 1.30 | 0.70-2.43 | 0.405 | |  |  |  |
| HBV infection (positive vs negtive) | 1.12 | 0.49-2.55 | 0.785 | |  |  |  |
| Cirrosis(Yes vs No) | 1.35 | 0.69-2.60 | 0.378 | |  |  |  |
| Child-Pugh class (B vs A) | 2.69 | 1.37-5.28 | 0.004 | | 2.19 | 0.98-4.88 | 0.055 |
| BCLC stage (C vs B) | 2.29 | 1.21-4.31 | 0.010 | | 2.14 | 1.01-4.51 | 0.047 |
| AFP（≥400ng/mL vs＜400ng/mL） | 2.89 | 1.34-6.23 | 0.007 | | 2.20 | 1.08-4.78 | 0.048 |
| AST（≥40U/L vs ＜40U/L） | 1.55 | 0.83-2.91 | 0.167 | |  |  |  |
| Tumor size(≥5cm vs ＜5cm) | 2.10 | 1.12-3.95 | 0.021 | | 3.25 | 1.47-7.19 | 0.003 |
| Tumor node (Multiple vs Single) | 1.62 | 0.81-3.25 | 0.176 | |  |  |  |
| Metastasis(Yes vs No) | 1.83 | 0.99-3.41 | 0.055 | | 1.01 | 0.48-2.11 | 0.976 |
| PVTT(Yes vs No) | 1.71 | 0.90-3.22 | 0.096 | | 1.30 | 0.64-2.63 | 0.467 |
| Surgery(Yes vs No) | 0.60 | 0.27-1.36 | 0.222 | |  |  |  |
| pTACE(＞2times vs ≤2 times) | 0.85 | 0.46-1.60 | 0.624 | |  |  |  |
| Treatment(TACE+Sor+ICIs vs TACE+Sor) | 0.26 | 0.13-0.53 | 0.000 | | 0.11 | 0.05-0.26 | 0.000 |

Abbreviations: PFS, progression-free survival; HCC, hepatocellular carcinoma; TACE, transarterial chemoembolization; pTACE, procedure before TACE refactory; Sor, sorafenib; ICIs，immune checkpoint inhibitors; ECOG-PS, Eastern Cooperative Oncology Group performance status; BCLC, Barcelona Clinic Liver Cancer; AFP, alpha-fetoprotein; AST, aspartate aminotransferase e; PVTT, portal vein tumor thrombus.

Table 6 Univariate and multivariate analyses of prognostic factors affecting OS in TACE-refractory advanced HCC

| Variables | Univariate Cox analysis | | | Multivariate Cox analysis | | |
| --- | --- | --- | --- | --- | --- | --- |
|  | HR | 95%*CI* | *P* value | HR | 95%*CI* | *P* value |
| Sex(male vs female) | 0.90 | 0.34-2.39 | 0.841 |  |  |  |
| Age(＜55 vs ≥55) | 1.10 | 0.53-2.11 | 0.880 |  |  |  |
| ECOG-PS (0-1 vs 2) | 1.19 | 0.59-2.43 | 0.623 |  |  |  |
| HBV infection (positive vs negtive) | 1.13 | 0.43-2.96 | 0.800 |  |  |  |
| Cirrosis(Yes vs No) | 1.31 | 0.62-2.77 | 0.477 |  |  |  |
| Child-Pugh class (B vs A) | 3.27 | 1.44-7.29 | 0.004 | 2.36 | 1.02-5.46 | 0.044 |
| BCLC stage (C vs B) | 4.72 | 2.02-11.02 | 0.000 | 3.88 | 1.56-9.60 | 0.003 |
| AFP（≥400ng/mL vs＜400ng/ml） | 2.83 | 1.25-6.45 | 0.013 | 2.50 | 1.06-5.90 | 0.037 |
| AST（≥40U/L vs ＜40U/L） | 1.33 | 0.65-2.71 | 0.436 |  |  |  |
| Tumor size(≥5cm vs ＜5cm) | 2.24 | 1.07-4.67 | 0.031 | 0.95 | 0.27-3.56 | 0.223 |
| Tumor node (Multiple vs Single) | 1.85 | 0.83-4.12 | 0.130 |  |  |  |
| Metastasis(Yes vs No) | 2.16 | 1.09-4.31 | 0.028 | 2.29 | 0.90-5.78 | 0.081 |
| PVTT(Yes vs No) | 1.64 | 0.80-3.35 | 0.176 |  |  |  |
| Surgery(Yes vs No) | 0.85 | 0.35-2.09 | 0.737 |  |  |  |
| pTACE(＞2times vs ≤2 times) | 0.79 | 0.39-1.59 | 0.515 |  |  |  |
| Treatment(TACE+Sor+ICIs vs TACE+Sor) | 0.41 | 0.19-0.84 | 0.016 | 0.24 | 0.11-0.55 | 0.001 |
| Albation(Yes vs No) | 0.28 | 0.12-0.64 | 0.003 | 0.29 | 0.12-0.75 | 0.010 |
| Radiotherapy(Yes vs No) | 0.67 | 0.30-1.48 | 0.321 |  |  |  |
| Second-line anti-angiogenesis agents(Yes vs No) | 0.73 | 0.36-1.47 | 0.378 |  |  |  |

Abbreviations: OS, overall survival; HCC, hepatocellular carcinoma; TACE, transarterial chemoembolization; Sor, sorafenib; ICIs，immune checkpoint inhibitors; ECOG-PS, Eastern Cooperative Oncology Group performance status; BCLC, Barcelona Clinic Liver Cancer; AFP, alpha-fetoprotein; AST, aspartate aminotransferase e; PVTT, portal vein tumor thrombus.

Table 7 Subgroup analysis of factor affecting PFS

|  | TACE+Sor | | TACE+Sor +ICIs | | *Test value* | *P* value |
| --- | --- | --- | --- | --- | --- | --- |
|  | Median | 95%*CI* | Median | 95%*CI* |  |  |
| BCLC stage |  |  |  |  |  |  |
| B | 9.4 | 6.8-11.9 | 21.2 | 15.5-27.00 | 11.726 | 0.001 |
| C | 4.8 | 3.3-6.2 | 10.8 | 7.5-14.1 | 9.084 | 0.003 |
| AFP（ng/ml） |  |  |  |  |  |  |
| ＜400 | 9.8 | 7.8-11.8 | 25.5 | 19.6-31.4 | 10.626 | 0.001 |
| ≥400 | 6.2 | 3.9-8.5 | 11.6 | 8.9-14.2 | 5.920 | 0.015 |
| Tumor size |  |  |  |  |  |  |
| ＜5cm | 10.0 | 7.34-12.6 | 21.6 | 14.4-28.8 | 8.504 | 0.004 |
| ≥5cm | 4.38 | 3.1-5.6 | 12.8 | 8.8-16.9 | 16.643 | 0.000 |

Abbreviations: PFS, progression-free survival; TACE, transarterial chemoembolization; Sor, sorafenib; ICIs, immune checkpoint inhibitors; BCLC, Barcelona Clinic Liver Cancer; AFP, alpha-fetoprotein.

Table 8 Subgroup analysis of factor affecting OS

|  | TACE+Sor | | TACE+Sor +ICIs | | *Test value* | *P* value |
| --- | --- | --- | --- | --- | --- | --- |
|  | Median | 95%*CI* | Median | 95%*CI* |  |  |
| BCLC stage |  |  |  |  |  |  |
| B | 20.3 | 13.3-27.4 | 30 | 23.5-36.6 | 3.300 | 0.069 |
| C | 7.3 | 4.0-10.5 | 13.5 | 9.7-17.3 | 4.854 | 0.028 |
| Child-Pugh class |  |  |  |  |  |  |
| A | 16.8 | 11.8-25.4 | 25.7 | 19.0-32.4 | 3.370 | 0.066 |
| B | 6.8 | 3.9-9.8 | 14.1 | 10.1-18.0 | 6.61 | 0.010 |
| Ablation |  |  |  |  |  |  |
| NO | 6.9 | 4.8-8.9 | 13.2 | 9.0-17.4 | 4.81 | 0.028 |
| Yes | 17.9 | 11.3-24.4 | 28.9 | 22.6-35.3 | 4.08 | 0.043 |

Abbreviations: OS, overall survival; BCLC, Barcelona Clinic Liver Cancer.

Figure legend

Figure 1: Flowchart shows patient selection.

Abbreviations: HCC, hepatocellular; BCLC, Barcelona Clinic Liver Cancer; ECOG-PS, Eastern Cooperative Oncology Group performance status; TACE, transarterial chemoembolization; Sor, sorafenib; ICIs, immune checkpoint inhibitors.

Figure 2 Kaplan–Meier analysis of PFS and OS.

Abbreviations: TACE, transarterial chemoembolization; Sor, sorafenib; ICIs, immune checkpoint inhibitors. PFS, Progression-free survival. OS, overall survival.

Figure 3 Subgroup analysis of PFS

Abbreviations: TACE, transarterial chemoembolization; Sor, sorafenib; ICIs, immune checkpoint inhibitors. PFS, Progression-free survival. OS, overall survival.

Figure 4 Subgroup analysis of OS

Abbreviations: BCLC, Barcelona Clinic Liver Cancer; AFP, alpha-fetoprotein.; TACE, transarterial chemoembolization; Sor, sorafenib; ICIs, immune checkpoint inhibitors. PFS, Progression-free survival. OS, overall survival.

Supplementary figure1: The median PFS and OS of patients receiving nivolumab or pembrolizumab in the TACE + Sor + ICIs group.

Abbreviations: TACE, transarterial chemoembolization; or, sorafenib; ICIs, immune checkpoint inhibitors. PFS, Progression-free survival. OS, overall survival.
